# Cardiac Arrest Mortality Across Time and Space: A National Analysis with Forecasts to 2035

**DOI:** 10.3390/jcm14144851

**Published:** 2025-07-08

**Authors:** Noman Khalid, Muhammad Abdullah, Sabrina Clare Higgins, Bilal Ahmad, Hasan Munshi, Mahnoor Hasnat, Muhammad Adil Afzal, Rajkumar Doshi, Rahul Vasudev, Shamoon E. Fayez, Julius M. Gardin, Julio A. Panza

**Affiliations:** 1 Department of Internal Medicine, New York Presbyterian Brooklyn Methodist Hospital, Brooklyn, NY 11215, USA; 2 Division of Clinical Research, Access Research Institute, Brooksville, FL 34613, USA; 3 Department of Internal Medicine, St. George’s University School of Medicine, Grenada FZ818, West Indies; sch.higgins@gmail.com; 4 Department of Public Health and Community Medicine, Shaikh Khalifa Bin Zayed Al Nahyan Medical and Dental College, Lahore 53720, Pakistan; bilalahmadjajja@gmail.com (B.A.); mahnoorhasnat39@gmail.com (M.H.); 5 Department of Internal Medicine, St. Joseph’s University Medical Center, Paterson, NJ 07503, USA; rmunshih@sjhmc.org (H.M.); r_afzalm@sjhmc.org (M.A.A.); 6 Department of Cardiovascular Medicine, St. Joseph’s University Medical Center, Paterson, NJ 07503, USA; raj20490@gmail.com (R.D.); vasudevr@sjhmc.org (R.V.); shamoonfa@sjhmc.org (S.E.F.); 7 Division of Cardiology, Rutgers New Jersey Medical School, Newark, NJ 07103, USA; jg1326@njms.rutgers.edu; 8 Department of Cardiology, Westchester Medical Center, Valhalla, NY 10595, USA; julio.panza@wmchealth.org

**Keywords:** cardiac arrest, mortality, epidemiology, public health, resuscitation

## Abstract

**Background**: Cardiac arrest remains a significant public health challenge with variable mortality trends across different demographics and regions, affecting healthcare planning and intervention strategies. We conducted this study to analyze cardiac arrest-related mortality trends from 1999 to 2023 and predict future trends up to 2035. **Methods**: This study analyzed data from 1999 to 2023, focusing on cardiac arrest as the primary cause of death (ICD-10: I46). Age-adjusted mortality rates (AAMRs) were standardized according to the 2000 U.S. Census. Joinpoint regression was utilized to calculate annual percentage change (APC), and an ARIMA model with Python 3.10 was used for mortality predictions. **Results**: A total of 365,608 cardiac arrest-related deaths were recorded in the USA from 1999 to 2023. There was a sharp decline in mortality rate until 2001 (APC: −10.35, *p* < 0.05), followed by a slowed decline until 2013 (APC: −2.91, *p* < 0.05), and then a gradual uptrend. Males exhibited a higher AAMR (5.8, 95% CI: 5.8–5.9) compared to females (4.2, 95% CI: 4.1–4.2). African Americans had the highest AAMR (8.9, 95% CI: 8.9–9), followed by Caucasians (4.8, 95% CI: 4.8–4.9) and American Indians (3.5, 95% CI: 3.3–3.7). The South region of the US had the highest AAMR, followed by the Northeast, Midwest, and West. Alabama exhibited the highest AAMR, followed by Nevada and Hawaii. Predictive analysis suggests a potential stable slow downtrend in mortality rates by 2035 (AAMR: 4.28, 95% CI: −1.8–10.4). **Conclusions**: The observed trends and future predictions underscore the importance of targeted public health interventions and healthcare planning to address cardiac arrest mortality.

## 1. Introduction

Cardiac arrest (Cardiac Arrest) is defined as the sudden termination of mechanical cardiac activity without evidence of the circulatory signs. If Cardiac Arrest results in attempts to restore circulation, this is known as Sudden Cardiac Arrest (SCA), whereas failure to restore circulation is known as Sudden Cardiac Death (SCD). Cardiac Arrest is commonly categorized as medical or external on the basis of etiology. Cardiac Arrest is broadly classified as Out-of-Hospital Cardiac Arrest (OHCA) and In-Hospital Cardiac Arrest (IHCA) in terms of site, where it has occurred. Cardiac Arrest is a significant healthcare burden on the U.S. economy as well as the individual affected by Cardiac Arrest. For instance, a study estimated that the cumulative average cost of OHCA in the U.S. was USD 33 billion each year. A huge chunk of this cumulative cost is due to advanced resuscitative measures, extensive diagnostic testing, intensive care unit stays, and interventional procedures if required.

According to the 2021 CARES registry, the United States (US) OHCA incidence is 92.3 per 100,000 at any age. Whilst IHCA incidence is extrapolated to 292,000 in the US [1]. 

It is important to analyze trends in Cardiac Arrest-related mortality, as it will aid us in determining whether mortality is improving or worsening with time. It will also help us to postulate or at least hypothesize the factors directly or indirectly affecting the mortality due to Cardiac Arrest. More importantly, the trends will help uncover disparities that are responsible for higher Cardiac Arrest mortality burden in some groups as compared to others. This can, in turn, help the healthcare authorities and health professionals in laying out a realistic framework for mitigating Cardiac Arrest mortality, keeping in mind the high-risk groups. Furthermore, bringing innovative yet effective measures into action will significantly reduce the healthcare burden of Cardiac Arrest-related mortality on the U.S. economy. Predicted future rates will help us see the trajectory we are heading in based on previous mortality. In this study we aim to analyze Cardiac Arrest mortality trends from 1999 to 2023 as well as predict future mortality rate trends using machine learning. 

## 2. Methods

This study utilized data spanning from 1999 through 2023, obtained from the Centers for Disease Control and Prevention’s Wide-Ranging Online Data for Epidemiologic Research (CDC WONDER) database. The data extracted focused on anonymized mortality records where Cardiac Arrest was listed as the main cause of death. The study population consisted of cases identified as cardiac arrest (CRC) as per the International Classification of Diseases, 10th Revision (ICD-10), specifically code I46. Data for population size, year, demographics, urban–rural classification, region, and states were extracted. Demographic information included sex, age, and race. NH White, NH Black or African American, Hispanic or Latino, NH American Indian or Alaskan Native, and NH Asian or Pacific Islander were the categories used for defining race. According to the NCHS Urban–Rural Classification scheme based on the 2000 U.S. census, the population was divided into large central metro, large fringe metro, medium metro, small metro, and non-metropolitan [2]. Regions were classified into Northeast, Midwest, South, and West according to the U.S. Census Bureau definitions.

IHCA is defined in the Utstein resuscitation registry reporting template as the delivery of chest compressions and/or defibrillation to patients already admitted to inpatient beds [3]. OHCA is defined as the cessation of functional cardiac mechanical activity along with an absence of systemic circulation, occurring outside of a hospital setting [4].

We assessed crude mortality rates (CMRs) and age-adjusted mortality rates (AAMRs) per 100,000 individuals and their 95% confidence intervals (CIs), correct to one decimal place, from 1999 to 2023 by year, age, sex, race, state, and urban–rural status to investigate national trends in Cardiac Arrest-related mortality. By dividing the total number of Cardiac Arrest-related deaths by the equivalent US population for that year, crude mortality rates were determined. AAMR was calculated by standardizing Cardiac Arrest-related deaths to the U.S. population in 2000. Direct standardization by 5-year age groups was applied to obtain AAMRs.

Age-adjusted mortality rate (AAMR) trends over time were evaluated using both linear regression and second-degree polynomial regression models to capture linear and potential curvilinear trends. For the linear model, the slope of the line provided an estimate of the annual change in AAMR. Polynomial models were used to visualize potential inflection points and nonlinear behavior in long-term trends.

To compare mean AAMRs between groups (e.g., sex, race, and census regions), we calculated mean ± standard error of the mean (SEM) for each subgroup (Supplementary Table S1). Two-sample independent t-tests were conducted for pairwise comparisons, and *p*-values were reported to assess statistical significance. A threshold of *p* < 0.05 was considered statistically significant. For regional and racial comparisons involving multiple groups, all pairwise comparisons were reported.

This study utilized the autoregressive integrated moving average (ARIMA) model to forecast future trends in cardiac arrest-related mortality. Data analysis and modeling were conducted using Python, a versatile programming language well-suited for handling large datasets and performing time series analysis. The ARIMA model was implemented through the statsmodels library, which offers robust statistical tools for autoregressive modeling and forecasting.

The ARIMA model combines three key components: the autoregressive (AR) component, which uses past values of the time series to predict future values; the integrated (I) component, which involves differencing the series to achieve stationarity; and the moving average (MA) component, which incorporates past forecast errors to improve prediction accuracy. Historical mortality data were analyzed to detect trends and ensure stationarity, applying differencing where necessary. The optimal model parameters (*p*, d, q) were selected based on model diagnostics and goodness-of-fit criteria, particularly the Akaike Information Criterion (AIC) and the Bayesian Information Criterion (BIC). The model’s performance was evaluated using standard error metrics, including Root Mean Square Error (RMSE) and Mean Absolute Percentage Error (MAPE), to ensure reliability and accuracy. Once validated, the ARIMA model was used to project cardiac arrest mortality rates up to the year 2035.

The Joinpoint Regression Program (Joinpoint V 5.1, National Cancer Institute, Bethesda, Maryland, USA) was used to calculate the annual average percent change (APC) with 95% CI in CMR and AAMR to measure national annual trends in Cardiac Arrest-related mortality [5]. This approach applies log-linear regression models when temporal variation occurs to identify significant changes in CMR or AAMR across time. If the slope representing the mortality change was statistically different from zero using a 2-tailed t-test, the APCs were considered rising or decreasing. This rise or decrease is across a time period that corresponds to the joinpoints calculated. A value of *p* < 0.05 was considered statistically significant in all cases.

In conducting this study, we followed the STROBE guidelines for reporting and were exempt from the requirement of informed consent or institutional review board approval due to the use of de-identified and publicly available data, consistent with the provisions of the Common Rule.

## 3. Results

**Figure 1:**
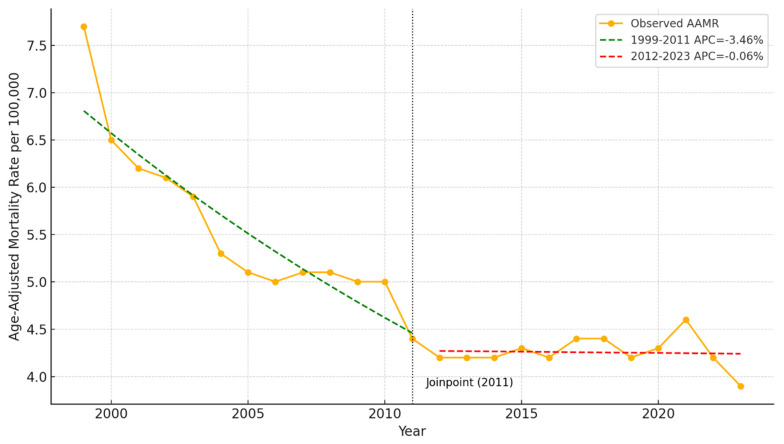
Cardiac arrest mortality rate (age adjusted) in the USA (1999–2023) with joinpoint regression.

A total of 365,608 Cardiac Arrest-related deaths were recorded among individuals aged ≥ 15 in the USA from 1999 to 2020. Figure 1 illustrates the age-adjusted mortality rate due to cardiac arrest in the USA from 1999 to 2023. A clear downward trend is observed over the period, with the highest rate recorded in 1999 at 7.7 deaths per 100,000 and the lowest in 2023 at 3.9 per 100,000. The quadratic trend line confirms a significant overall decline, particularly in the early 2000s, followed by a plateau in recent years. Confidence intervals show relatively stable variability across the timeline.

**Figure 2:**
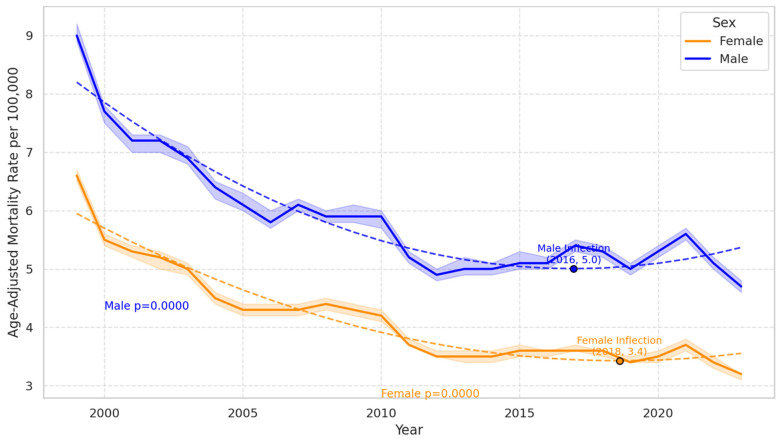
Cardiac arrest mortality rate by sex with polynomial regression (1999–2023).

Gender-based analysis revealed a consistently higher mortality rate in males compared to females. Figure 2 presents the age-adjusted cardiac arrest mortality rates by sex in the USA from 1999 to 2023. Both males and females exhibited significant declines over time (*p* < 0.0001 for each). Males consistently had higher mortality rates than females throughout the period. An inflection point is observed for males in 2016 at a rate of 5.0 per 100,000 and for females in 2018 at 3.4 per 100,000, indicating a slowing in the decline. Despite the overall downward trend, recent years show a slight uptick, particularly among males. In females, the APC showed a notable decrease of −10.58% between 1999 and 2001, followed by a slower decline (APC: −3.00%) until 2013, and then a marginal decrease (APC of −0.30%) through to 2020. The AAMR in females decreased from 6.6 in 1999 to 3.5 in 2020. In contrast, males also exhibited a similar pattern of decline with an APC of −10.19% from 1999 to 2001 and −2.85% from 2001 to 2013, followed by a slight increase of 0.60% from 2013 to 2020. The AAMR for males decreased from 9.0 in 1999 to 5.2 in 2020 (Table 1, Figure 2).

**Figure 3:**
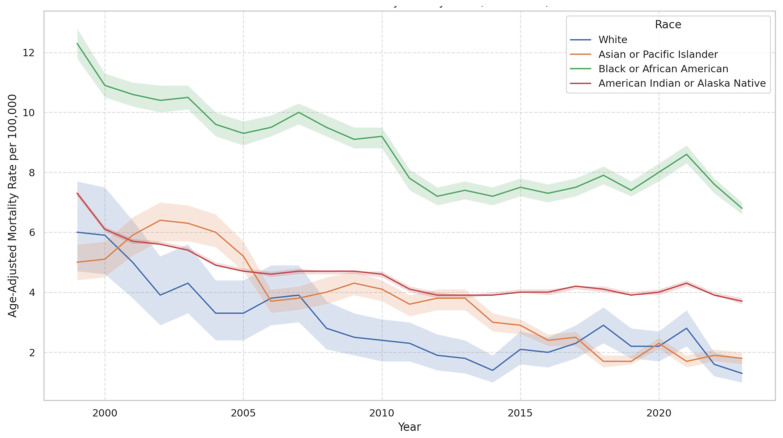
Cardiac arrest mortality rate by race (1999–2023) with 95% CI.

The mortality rates varied considerably across different racial groups. Figure 3 shows the age-adjusted cardiac arrest mortality rates by race in the USA from 1999 to 2023. Black or African American individuals consistently experienced the highest mortality rates across the entire period, though rates declined from over 12 per 100,000 to around 7 per 100,000. In contrast, White, Asian or Pacific Islander, and American Indian or Alaska Native populations exhibited lower and more convergent rates over time. All racial groups show a general downward trend. Black or African American females showed a marked decrease in mortality rates from 1999 to 2005, followed by a brief increase and then another decrease until 2013, but a slight increase was noted from 2013 to 2020. White females experienced a substantial decline from 1999 to 2001 and then a steady rate until 2020. Hispanic or Latino females saw a significant decrease from 1999 to 2002, followed by a slight increase through to 2020 Asian or Pacific Islander females exhibited a continuous decline, particularly after 2012 (Table 2).

In contrast, Black or African American males displayed a significant decrease in mortality rates until 2014, followed by an increase until 2020. White males also saw a notable decrease until 2012, followed by a slight increase. Hispanic or Latino males experienced a sharp decline from 1999 to 2001 and then a gradual increase. American Indian or Alaskan Native males showed a decrease until 2014 but a substantial increase thereafter (Table 2).

**Figure 4:**
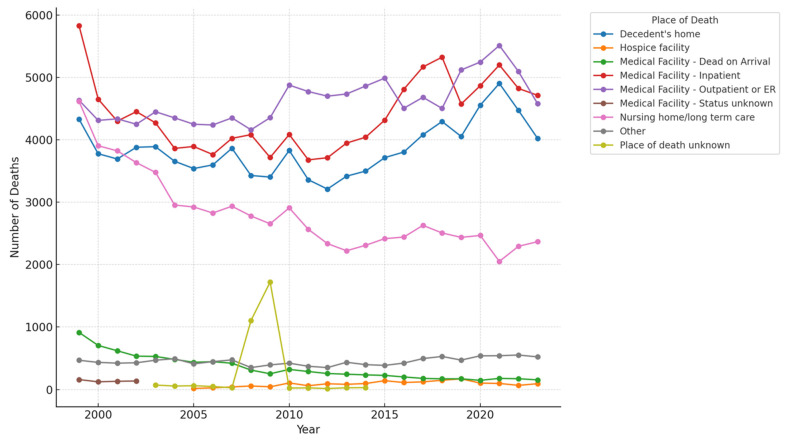
Cardiac arrest deaths by place (1999–2023) (total number).

The place of death having the highest number of deaths in 1999 was inpatient medical facilities, which showed a decline over the years till 2010 but started to steeply rise till 2018. Moreover, the highest number of deaths in 2023 was also in inpatient medical facilities. Outpatient medical facility or ER deaths followed closely with almost the same number of deaths as in inpatient medical facilities in 2023. The least number of deaths were reported in unknown places of death, followed closely by deaths in hospice facilities as depicted in Figure 4.

**Figure 5:**
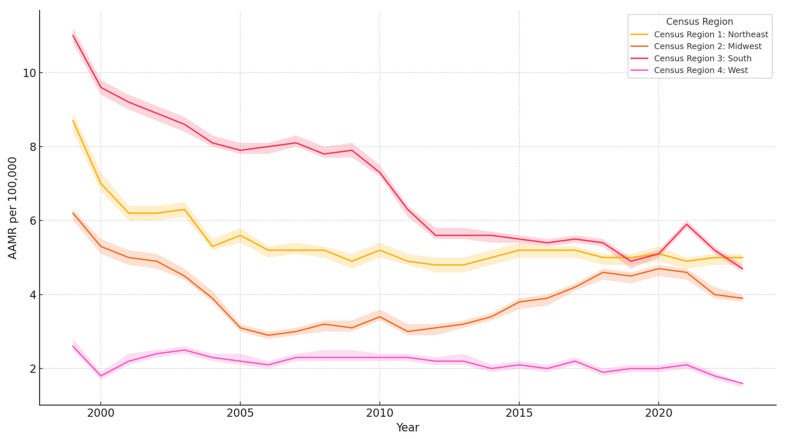
Cardiac arrest AAMR by census region (1999–2023).

In terms of geographic distribution, the South region recorded the highest AAMR at 6.9, with the Northeast following at 5.4, and the West exhibiting the lowest rate at 2.2 (Table 3). The South region recorded the greatest decline in AAMR, whereas the West region recorded almost stagnant AAMR over the period of our study, as shown in Figure 5.

**Figure 6:**
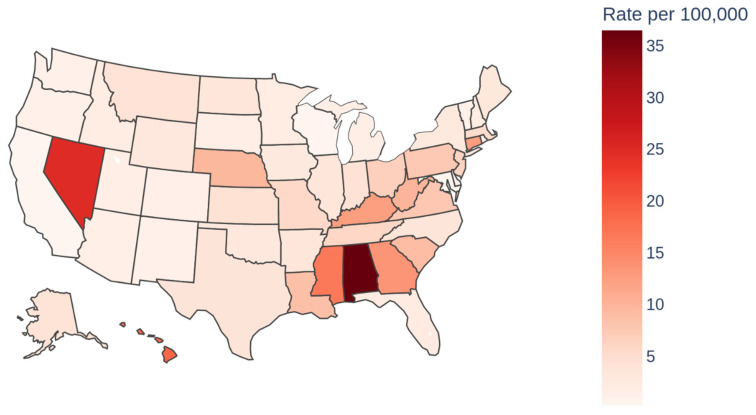
Cardiac arrest mortality by state for 2023 (AAMR).

At the state level, Alabama, Nevada, and Hawaii were observed to have the highest AAMRs, with Alabama leading at 36.5 (Table 3) (Figure 6).

Analyzing trends based on urbanization levels revealed that nonmetropolitan areas experienced the highest AAMR at 8.7, in contrast to large fringe metropolitan areas, which had the lowest AAMR at 3.1.

**Figure 7:**
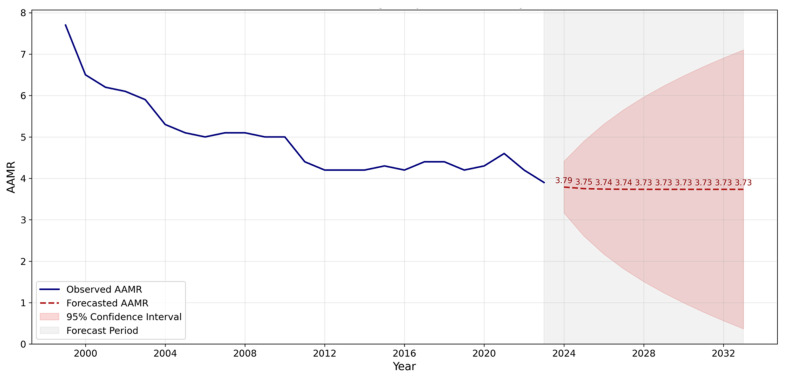
ARIMA forecast of age-adjusted mortality rate (AAMR).

The time-series analysis, depicted in the graph, illustrates the historical age-adjusted rate (AAMR) of Cardiac Arrest-related mortality from 1999 to 2023 alongside the forecasted rates extending up to 2035. Historically, there has been a noticeable decline in the AAMR from 7.7 in 1999 to 5.6 in 2020. In Figure 7, the AAMR calculated from 2020 to 2023 shows a steady trend in which it remains level at 7 per 100,000. The ARIMA model projects a slight decline in the coming years, although not as steep as seen in the past. The 95% confidence intervals, represented by the shaded areas, widen over time, reflecting growing uncertainty in the projections as we move further from the historical data. Notably, the model’s confidence intervals suggest increased variability in the AAMR, underscoring the potential for broader fluctuation in future rates. These findings highlight a continued but slowing improvement in Cardiac Arrest mortality outcomes over the next fifteen years, as shown in Figure 7.

## 4. Discussion

### 4.1. Year-Wise Mortality Rate

Across two decades the rate of mortality from Cardiac Arrest has continuously decreased. Innovations and protocols implemented from 2001 to 2013 have likely led to a significant, constant decline in mortality. These innovations include Minimally Interrupted Cardiac Resuscitation (MICR), which emphasizes continuous chest compressions with minimal interruptions, delaying endotracheal intubation and promptly administering epinephrine. A study shows that survival rates increased from 7.5% to 13.9% in OHCA cases after implementing MICR [6]. Notably, the effects of the AHA’s introduction of the Chain of Survival concept in 2000 likely kickstarted the decline [7]. Followed shortly by impedance threshold devices to maximize blood flow in 2005 and public engagement with AHA’s 2005 CPR guidelines emphasizing Hands-Only CPR, allowing bystanders to participate in resuscitation without mouth-to-mouth exposure [8,9]. The period from 2005 to 2008 saw targeted temperature management become the AHA’s standard of care with the goal of improving neurologic outcomes post-Cardiac Arrest by therapeutic hypothermia, although this does not improve mortality [10]. Combined, these factors made strides in improving outcomes. Interestingly, mobile phone applications like GoodSAM enable early CPR and AED use by alerting nearby trained volunteers to Cardiac Arrest events. Studies have shown that such measures can double the chance of survival to hospital discharge [11,12]. Moreover, recent advances in the treatment modalities of precipitating factors of cardiac arrest have also significantly contributed to declining mortality. For instance, modern revascularization techniques for coronary heart disease, the leading cause of cardiac arrest, such as optical coherence tomography- and intravascular ultrasound-guided PCI, minimally invasive coronary artery bypass grafting and hybrid revascularization, have led to a lower incidence of cardiac arrest due to coronary heart disease [13]. Likewise, other major advancements such as implantable cardioverter-defibrillators for treating arrhythmias, advanced electrophysiological mapping for improving catheter ablation procedures, and wearable monitoring devices facilitating early intervention have also played a crucial role in reducing cardiac arrest-related mortality [13]. However, 2013–2020 saw a stabilizing trend, which warrants further investigation to elucidate underlying causes. This question’s multifaceted nature would require specific analyses that are outside the scope of this study. Nevertheless, we can hypothesize that it may be due to having reached saturation in our current understanding and treatment of cardiac arrest, resulting in reaching a plateau in trends in survival irrespective of guidelines. This hypothesis is partly supported by findings of Holmberg et al., in which they found no significant immediate change in survival following the introduction of guidelines. Their sensitivity analysis showed that the survival trend remained stagnant from 2010 till 2015 and also post-2015 in hospitals providing data to the Get With The Guidelines–Resuscitation (GWTG-R) registry during that period [14]. Other factors, such as increased burden of comorbidities among patients with IHCA and a younger population having IHCA, may also contribute to the reversal in declining mortality from 2013 onwards [15].

The data from many registries recording Cardiac Arrest-related mortality data aligns with our findings. Data from the CARES registry shows that risk-adjusted survival rates for OHCA increased from 5.7% in 2005–2006 to 8.3% in 2012, which can be attributed to enhanced bystander CPR rates and increased use of automated external defibrillators (AEDs) [16]. The data from the GWTG-R registry regarding IHCA also corroborates our findings. According to this registry, survival to discharge after IHCA improved from 13.7% in 2000 to 22.3% in 2009 [17]. However, it was noted that the annual increase in survival was more pronounced in major teaching hospitals as compared to minor teaching hospitals [18]. According to a study based on the National Inpatient Sample database, there was greater than a 10% improvement in IHCA-related survival rates after risk adjustment. This can be largely attributed to improvements in PEA-asystole survival and, to some degree, to ventricular tachycardia/ventricular fibrillation survival [15].

An important point to note is a significant rise in mortality in inpatient medical facilities from 2010 to 2018. This can be attributed to the ineffectiveness of the 2010 and 2015 resuscitation guidelines, mainly due to the lack of implementation of these guidelines for effectively addressing the complexities of IHCA management [14]. Moreover, a significant proportion of IHCA cases present with nonshockable rhythms, which have a poorer prognosis as compared to shockable rhythms. The prevalence of nonshockable rhythms in IHCA patients is alarmingly high at 81%, leading to poor outcomes [19]. Furthermore, comorbid conditions such as diabetes, hypertension, and coronary artery disease have become more prevalent among patients experiencing IHCA, complicating resuscitation efforts and post-arrest care [15]. Another interesting factor is the impact of the timing of IHCA on the prognosis of the patient. IHCA events occurring during nighttime hours and weekends are associated with higher mortality rates [20].

### 4.2. Gender-Specific Mortality Trends

Among genders, overall males showed a higher mortality rate than females throughout the study period. Both genders saw a decline in mortality from 1999 to 2013. However, males saw a minimal, gradual increase in mortality from 2013 onwards compared to females. Gender disparities regarding OHCA are well documented, emphasizing that females are historically older than males at the time of Cardiac Arrest but also respond better to resuscitation efforts [21]. Males have a higher rate of cardiovascular disease (CVD) mortality than females; therefore, they likely have a higher rate of Cardiac Arrest [22]. Although the increase from 2013 can be correlated to CVD, there may be other underlying factors that need to be further explored. Social determinants of health may be the cornerstone to understanding this discrepancy.

### 4.3. Overall Racial and Gender-Specific Racial Mortality Trends

African Americans experienced a mortality rate nearly twice that of Caucasians, while Hispanics or Latinos curiously showed the lowest mortality rates. Males of all races, except for Hispanics or Latinos, observed a decrease in mortality until the period from 2012 to 2014, which was then followed by a drastic increase in mortality rates among African American and American Indian males. Females across all races experienced a steady decrease in mortality overall, except for African American females, who saw an increase in mortality starting from 2013.

This drastic change in mortality questions what changed socioeconomically after 2012 to impact these specific demographics but would require in-depth analysis not in the scope of this study. Hispanics or Latino male and female populations had a gradual increase in mortality across the decades. This is interesting considering that overall, Hispanics and Latinos had the lowest mortality among racial groups. It could be postulated that this trend is observed due to this group seeking out medical assistance due to cultural beliefs, health education, and access to insurance, albeit governmental or private.

It could be that minority groups might have worse outcomes, as they have multiple barriers to healthcare and have a higher SVI. SVI domains include socioeconomic status, household composition and disability, minority status and language, housing, and transportation. Interestingly, the 2023 Census Bureau statistics show that 19.1% of the population is Hispanic or Latino, while only 13.6% is African American or Black [23]. Yet the CDC states that African American males and females are more likely to die due to OHCA than their Caucasian counterparts [24]. African American and Hispanic populations have higher vulnerabilities correlating to worse outcomes, as illustrated during the COVID-19 pandemic [25]. Predisposition to hypertension, obesity, and diabetes compounded by selective access to high-quality health care are considerations when approaching policy change and public health interventions to decrease Cardiac Arrest mortality in these groups [26].

### 4.4. Region-Specific Mortality Trends

Southern regions of the USA saw the highest AAMR, with the West having the lowest AAMR. This is perplexing since the West has the highest total hospitalization cost and hospital charges. However, it could be related to procedural costs, imaging, consultations and diagnostic and therapeutic procedures (PCI, therapeutic hypothermia, and implantable cardioverter defibrillator) [27]. Identifying the specific driving factors for such an increase is an important area for further research. Differences in hospital resources, availability of specialized care, and post-resuscitation services may impact survival rates and costs [28]. Fluctuation in mortality is not unexpected, as factors like access to public AEDs, bystander involvement, and EMS response times are variable regionally. Prior studies have suggested that protocol variations such as hypothermia and coronary angiography may be implicated in variation [29]. Interestingly, Alabama was implicated in prior studies as well as ours as having the highest mortality rates for Cardiac Arrest, questioning state governance and public health organizations [29]. Correlations between variables listed would need extensive cohort studies and analyses of their own to truly be valuable statistics. Efforts to standardize healthcare, facilitate access to advanced treatments, and implement region-specific strategies may aid in lowering mortality rates nationwide.

Non-metropolitan regions showed mortality rates 2.8 times higher than metropolitan. This illustrates why public health efforts for bystander CPR education and AED accessibility are crucial, particularly in far-flung locations.

### 4.5. Future Predictions

Future predictions for mortality extrapolate a stable trend till 2035 similar to that illustrated in the second decade. A stable extrapolation affirms that new guidelines and public health awareness to engage bystanders have made meaningful contributions to a favorable mortality trend. Such implementation in the future, combined with continued public health drives to engage bystanders in events of Cardiac Arrest will further improve mortality outcomes.

This study spans over two decades to reveal impactful long-term trends in Cardiac Arrest outcomes. It delves deep into demographic and geographical variations, highlighting disparities across gender, racial groups, and regions, which is crucial for forming targeted public health interventions. Its methodology, utilizing advanced statistical techniques like Joinpoint Regression and ARIMA modeling, allows for a detailed examination of shifts in mortality rates in the past and future predictions. By focusing on ethical considerations and utilizing de-identified data, the study also ensures adherence to ethical research standards.

The study has some limitations, including potential biases from using retrospective CDC WONDER database data and the simplification of complex factors through statistical methods. It may not fully capture the intricate effects of socio-economic status, healthcare access, and individual behaviors on mortality trends. Additionally, the ARIMA model predicts based on past trends and does not account for future changes in policies or any pandemic like COVID-19. These constraints highlight the necessity for cautious interpretation of results and ongoing research to refine projections and improve public health strategies. The study does not have survivor information like the time from which the CPR was started, which has a significant impact; reason for death; cardiac vs. non-cardiac mortality; and neurological status, even if survived.

## 5. Conclusions

In conclusion, the study highlights a general decline in Cardiac Arrest mortality rates, attributing this positive trajectory to medical innovations, improved resuscitation techniques, and public health interventions developed over the past two decades. However, the slight increase in mortality rates among certain demographics post-2013 calls for a refined approach in addressing the socioeconomic factors and healthcare disparities that continue to influence Cardiac Arrest outcomes. Moreover, these socioeconomic factors and healthcare disparities must be promptly addressed to further increase the rate of decline in Cardiac Arrest mortality rates, since the decline projected by the ARIMA model is uncertain to some extent, which largely depends upon whether these crucial socioeconomic factors and healthcare disparities are adequately addressed. The benefit of projecting the trend to 2035 is to highlight that, given the current strategies being employed to reduce Cardiac Arrest mortality rates, the decline projected to 2035 is not quite significant, which indicates that the current measures to mitigate Cardiac Arrest mortality are very likely to be insufficient, at least to 2035. The study’s insights into the differential impacts of Cardiac Arrest across diverse populations and regions emphasize the need for tailored strategies that address the unique challenges and vulnerabilities of each group. As we move forward, healthcare professionals, policymakers, and public health advocates must collaborate closely to build on the progress achieved and tackle the persisting gaps in Cardiac Arrest mortality prevention and care.

## Figures and Tables

**Table 1:** Age-adjusted mortality rate by gender.

Gender	Years/Range (AAMR)	APC
Female	1999 (6.6)–2001 (5.2)	−10.58 *
	2001 (5.2)–2013 (3.5)	−3
	2013 (3.5)–2020 (3.5)	−0.3
Male	1999 (9.0)–2001 (7.1)	−10.19 *
	2001 (7.1)–2013 (5.0)	−2.85 *
	2013 (5.0)–2020 (5.2)	0.6

Asterisk (*) indicates that the result is statistically significant with a *p*-value < 0.05. AAMR: age-adjusted mortality rate; APC: annual percentage change.

**Table 2:** Annual percentage change of cardiac arrest mortality by race and gender.

Race	Gender	Years/Range	APC
Asian or Pacific Islander	Female	1999–2012	3.08
		2012–2020	−9.24 *
Black or African American	Female	1999–2005	−3.49 *
		2005–2008	1.52
		2008–2013	−6.11 *
		2013–2020	0.85
White	Female	1999–2001	−11.79 *
		2001–2013	−2.85
		2013–2020	0.03
Hispanic or Latinos	Female	1999–2002	−16.95 *
		2002–2020	0.39
Black or African Americans	Male	1999–2014	−4.05 *
		2014–2020	1.8
White	Male	1999–2001	−10.92 *
		2001–2012	−2.81 *
		2012–2020	0.36
Hispanic or Latinos	Male	1999–2001	−25.49 *
		2001–2020	0.84
American Indian or Alaskan Native	Male	1999–2014	−6.25 *
		2014–2020	6.91
Asian or Pacific Islander	Male	1999–2002	8.6
		2002–2020	−6.43 *

Asterisk (*) indicates that the result is statistically significant with a *p*-value < 0.05. APC: annual percentage change.

**Table 3:** Age-adjusted mortality rates according to US census regions, US states, and urbanization.

Category	Subcategory	Rank	AAMR
US Census Region Trends	South	Highest	6.9
	Northeast	2nd Highest	5.4
	West	Lowest	2.2
USA States Trends	Alabama	Highest	36.5
	Nevada	2nd	25
	Hawaii	3rd	18.7
Urbanization	Nonmetro	Highest	8.7
	Large Fringe Metro	Lowest	3.1

AAMR: age-adjusted mortality rate.

## Data Availability

The data presented in this study are publicly available from the Centers for Disease Control and Prevention Wide-ranging Online Data for Epidemiologic Research (CDC WONDER) database. This resource is publicly accessible at: https://wonder.cdc.gov (accessed on 24 May 2024) No new data were created or collected specifically for this study.

## Supplementary Materials

The following supporting information can be downloaded at: https://www.mdpi.com/article/10.3390/jcm14144851/s1, Supplementary Table S1: AAMR by sex and pairwise *p*-value, AAMR by race and pairwise *p*-value.
