# Supplementary material for: Cardiac Arrest Mortality Across Time and Space: A National Analysis with Forecasts to 2035
Source: J Clin Med. 2025 Jul 8;14(14):4851. doi: 10.3390/jcm14144851 (PMC12295983; doi:10.3390/jcm14144851)
Supplement: Supplementary file 1 [file jcm-14-04851-s001.zip › jcm-3674555-supplementary.pdf]

Supplementary Table S1: AMR by sex and pairwise p-value, AAMR by race and pairwise p-value

Group	Mean AAMR	SEM	Pairwise-p value
Female	4.15	0.166	0.0000000462
Male	5.87	0.208	

Group	Mean AAMR	SEM
American Indian or Alaska Native	4.6	0.171
Asian or Pacific Islander	3.72	0.305
Black or African American	8.76	0.293
White	2.96	0.259

	American Indian or Alaska Native	Asian or Pacific Islander	Black or African American	White
American Indian or Alaska Native				
Asian or Pacific Islander	0.0153		5.62E-16	
Black or African American	2.00E-16			

White	3.11E-06	0.0636	1.43E-19	
